# Recent Advances on Immunohistochemistry and Molecular Biology for the Diagnosis of Adnexal Sweat Gland Tumors

**DOI:** 10.3390/cancers14030476

**Published:** 2022-01-18

**Authors:** Nicolas Macagno, Pierre Sohier, Thibault Kervarrec, Daniel Pissaloux, Marie-Laure Jullie, Bernard Cribier, Maxime Battistella

**Affiliations:** 1 French Network of Rare Skin Cancers, CARADERM, France; pierre.sohier@aphp.fr (P.S.); Thibault.Kervarrec@yahoo.fr (T.K.); marie-laure.jullie@chu-bordeaux.fr (M.-L.J.); bernard.cribier@chru-strasbourg.fr (B.C.); maxime.battistella@aphp.fr (M.B.); 2 Department of Pathology, APHM, Timone University Hospital, 13005 Marseille, France; 3 Marmara Institute, Aix-Marseille University, INSERM, U1251, MMG, DOD-CET, 13005 Marseille, France; 4 Faculté de Médecine, Paris Centre Santé, University of Paris, 75006 Paris, France; 5 Department of Pathology, Cochin Hospital, AP-HP, AP-HP Centre-Université de Paris, 750014 Paris, France; 6 Department of Pathology, Université de Tours, Centre Hospitalier Universitaire de Tours, 37000 Tours, France; 7 Team: Biologie des Infections à Polyomavirus, UMR INRA ISP 1282, Université de Tours, 37000 Tours, France; 8 Department of Biopathology, UNICANCER, Léon Bérard Center, 69008 Lyon, France; Daniel.pissaloux@lyon.unicancer.fr; 9 INSERM 1052, CNRS 5286, Cancer Research Center of Lyon, Lyon University, 69008 Lyon, France; 10 Department of Pathology, Haut-Lévêque Hospital, CHU de Bordeaux, 33600 Bordeaux, France; 11 Dermatology and Dermatopathology, Faculty of Medicine, University Hospital, University of Strasbourg, 67000 Strasbourg, France; 12 Department of Pathology, Saint-Louis University Hospital, 75010 Paris, France

**Keywords:** adenoid cystic carcinoma, *NFIB*, *MYB*, *MYBL1*, mixed tumor, chondroid syringoma, *PLAG1*, cylindroma, spiradenoma, *CYLD*, *ALKP1*, hidradenoma, *MAML2*, myoepithelioma, *EWSR1*, *FUS*, poroma, porocarcinoma, poroid hidradenoma, *YAP1*, *NUTM1*, secretory carcinoma, *ETV6*, *NTRK3*, tubular adenoma, syringocystadenoma papilliferum, *HRAS*, *BRAF*

## Abstract

**Simple Summary:**

Cutaneous sweat gland tumors form an extremely diverse and heterogeneous group of neoplasms that show histological differentiation to the sweat apparatus. Due to their rarity, wide diagnostic range, and significant morphological overlap between entities, their accurate diagnosis remains challenging for pathologists. Until recently, little was known about the molecular pathogenesis of adnexal tumors. Recent findings have revealed a wide range of gene fusions and other oncogenic factors that can be used for diagnostic purposes and, for some, can be detected by immunohistochemistry. Among other organs containing exocrine glands, such as salivary glands, breasts, and bronchi, most of these biomarkers have been reported in homologous neoplasms that share morphological features with their cutaneous counterparts. This review aims to describe these recent molecular and immunohistochemical biomarkers in the field of sweat gland tumors.

**Abstract:**

Cutaneous sweat gland tumors are a subset of adnexal neoplasms that derive or differentiate into the sweat apparatus. Their great diversity, rarity, and complex terminology make their pathological diagnosis challenging. Recent findings have revealed a wide spectrum of oncogenic drivers, several of which are of diagnostic interest for pathologists. Most of these molecular alterations are represented by gene fusions, which are shared with other homologous neoplasms occurring in organs containing exocrine glands, such as salivary and breast glands, which show similarities to the sweat apparatus. This review aims to provide a synthesis of the most recent immunohistochemical and molecular markers used for the diagnosis of sweat gland tumors and to highlight their relationship with similar tumors in other organs. It will cover adenoid cystic carcinoma (*NFIB*, *MYB,* and *MYBL1* fusion), cutaneous mixed tumor (*PLAG1* fusion), cylindroma and spiradenoma and their carcinomas thereof (NF-κB activation through *CYLD* inactivation or *ALKP1* hotspot mutation), hidradenoma and hidradenocarcinoma (*MAML2* fusion), myoepithelioma (*EWSR1* and *FUS* fusion), poroma and porocarcinoma (*YAP1*, *MAML2,* and *NUTM1* fusion), secretory carcinoma (*ETV6*, *NTRK3* fusion), tubular adenoma and syringo-cystadenoma papilliferum (*HRAS* and *BRAF* activating mutations). Sweat gland tumors for which there are no known molecular abnormalities will also be briefly discussed, as well as potential future developments.

## 1. Introduction

Benign and malignant cutaneous adnexal tumors are rare [1]. They span a wide variety of different diagnoses, classified according to their apocrine, eccrine, follicular, and sebaceous differentiation. Similarly, these tumors have a wide range of prognoses, including benign tumors, low-grade locally recurring tumors, and aggressive carcinomas prone to metastases.

An update of the nosology of tumors of cutaneous appendages has been proposed by the fourth edition of the WHO classification of cutaneous neoplasms [2]. At the time of its publication, many clinicopathological entities lacked a specific and robust diagnostic hallmark, but recent discoveries have revealed new biomarkers with high diagnostic value. Many of these markers are related to specific molecular alterations demonstrated in these neoplasms.

Most skin carcinomas, especially basal and squamous cell carcinomas, exhibit an ultraviolet mutational signature and a high tumor mutational burden, which are considered key oncogenic events in their initiation and progression [3,4]. On the contrary, adnexal tumors, which occur primarily deep in the skin, are less prone to UV exposure. Adnexal tumors are, therefore, prone to stochastic but recurrent genetic alterations, such as missense mutations and oncogenic gene fusions. Interestingly, many of these molecular alterations can also be observed in other organs containing exocrine glands, i.e., salivary glands, lacrimal glands, lung, pancreas, and breast. Therefore, cutaneous adnexal tumors are frequently analogous to extracutaneous tumors with similar morphological, phenotypic, and molecular characteristics but with different, sometimes confusing, terminologies. For example, in salivary glands, the term “cylindroma” has been used as a synonym of adenoid cystic carcinoma and, while adenoid cystic carcinoma cases of the skin are analogous to their salivary counterparts, with recurrent fusion involving the *MYB* gene, skin cylindromas represent a distinct tumor entity with frequent *CYLD* mutation. Furthermore, some of these biomarkers are related to a whole group of adnexal neoplasms since they are detected in benign and malignant cases, while some evidence suggests a multistep progression from a benign precursor with a specific molecular background to their malignant counterpart. Histopathological classifications attempted to separate the wide variety of adnexal neoplasms based on their differentiation to specific parts of the skin appendages, including apocrine, eccrine, follicular, sebaceous, and multilineage differentiation. The cell of origin remains the subject of debate and investigation for many neoplasms. The difficulty in determining the cell of origin is related in part to the biology of many fusion-driven sweat gland tumors. Indeed, chimeric fusion proteins confer a new, often aberrant, histological phenotype that lacks a physiological cellular counterpart to correlate with for classification purposes. Hopefully, most of these gene fusions are highly specific in each morphological context, being mostly restricted to a single subtype of tumor.

This review aims to provide a synthesis of the most recent immunohistochemical (Table 1) and molecular markers (Table 2) used for the diagnosis of cutaneous adnexal neoplasms with sweat gland differentiation.

We will review, in alphabetical order, adenoid cystic carcinoma (*NFIB* and *MYB* fusion), mixed tumor (*PLAG1* fusion), cylindroma and spiradenoma (NF-κB activation through *CYLD* inactivation or *ALKP1* hotspot mutation), hidradenoma (*CRTC1/3* and *MAML2* fusion), myoepithelioma (*EWSR1* and *FUS* fusion), poroma (*YAP1* and *NUTM1* fusion), secretory carcinoma (*ETV6* and *NTRK3* fusion), tubular adenoma and syringocystadenoma papilliferum (*HRAS* and *BRAF* activating mutations). For each entity, data regarding the respective homologous extracutaneous tumors will be mentioned.

Finally, sweat gland tumors lacking known recurrent molecular abnormalities will be briefly discussed in the last paragraph of this review.

## 2. Sweat Gland Neoplasms with Recent Advances in Their Diagnosis

### 2.1. Adenoid Cystic Carcinoma

**Figure 1:**
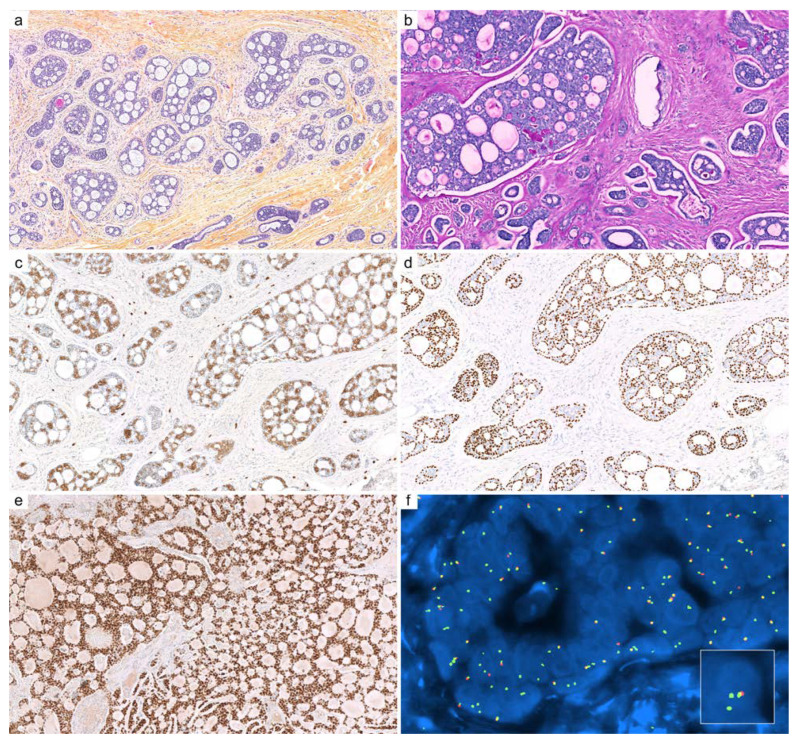
Cutaneous adenoid cystic carcinoma (ACC): (**a**) ACC is arranged in various cribriform aggregates and ductal structures (HPS, ×100); (**b**) periodic acid-Schiff (PAS) reaction highlights entrapped basement membrane material within aggregates (HPS, ×200); (**c**) c-KIT (CD117, ×200) immunohistochemistry stains luminal cells; (**d**) immunostaining for p63 highlights outer myoepithelial cells with a discontinuous pattern (×200); (**e**) SOX10 immunohistochemistry is intensely and diffusely positive in the nuclei of ACC (×200); (**f**) fluorescence in situ hybridization (FISH, break-apart probe) demonstrates a clonal rearrangement of *MYB* in neoplastic cells (×1000).

General features: Adenoid cystic carcinoma (Figure 1) is a rare cutaneous adnexal carcinoma, characterized histologically by an epithelial and myoepithelial phenotype. Most cases demonstrate *MYB::NFIB* or *MYBL1::NFIB* gene fusions [5,6,7].

Related terminology: The designation adenoid cystic carcinoma is used regardless of the location considered. Confusingly, the term ‘cylindroma’ has historically been used to refer to salivary gland adenoid cystic carcinoma.

Immunohistochemistry: Most ACCs are positive for SOX10, with an intense and diffuse staining pattern [8,9,10,11]. Calponin, SMA, PS100, and p63 reveal myoepithelial differentiation, while c-KIT (CD117), EMA, CEA, and various cytokeratins label epithelial cells that line the ducts [12,13,14]. Immunostaining for p63 is often discontinuous compared to SOX10, which is more intense and diffuse [15]. Furthermore, c-KIT (CD117), MYB, and GATA3 are expressed in 78–100% [13,16,17], 90% [14], and 20% of cases [14], respectively, but all lack specificity [12,18].

Molecular biology: Fusions of MYB or MYBL1 with NFIB are detected in 40–97% of adenoid cystic carcinomas with very high specificity [5,6,12,16,19,20]. The most frequent fusions are *MYB::NFIB* fusion (73–83%) and *MYBL1::NFIB* (20–23%) [16,21,22,23,24], with detection of gene rearrangement in both myoepithelial and ductal epithelial cells [22]. Up to 83% of cutaneous cases demonstrate *MYB* rearrangement. Extracutaneous cases lacking these recurrent alterations have been reported with alternative fusion partners (*ACTN1*) and amplification of *MYB* [25]. MYB overexpression can also occur in the absence of detectable alteration, suggesting alternative unknown mechanisms in a subset of cases. MYB proto-oncogene, transcription factor (*MYB*), and MYB proto-oncogene like 1 (*MYBL1*) are genes mapped on chromosomes 6 and 8, respectively. *MYB* and *MYBL1* rearrangements and amplification cause cell proliferation [26] through the dysregulation of MYB target genes, which are involved in the control of cell cycle: *NSR*, *MET*, *EGFR*, *IGF1R*, and *IGF2* (89). The NOTCH pathway genes are commonly altered in advanced adenoid cystic carcinoma and can be targeted by specific therapies [27].

Discordant or controversial data: Contrary to the high specificity reported by numerous studies [5,6,12,16,19,20], *MYB::NFIB* fusions have been reported in tumors diagnosed as wild-type *CYLD* cutaneous cylindroma in a single report [28], but this finding was not further confirmed [29]. Since cylindroma and adenoid cystic carcinoma show significant histopathological overlap, the possibility of *MYB* fusion in bona fide cutaneous cylindroma is highly questionable.

### 2.2. Cutaneous Mixed Tumor (Chondroid Syringoma)

**Figure 2:**
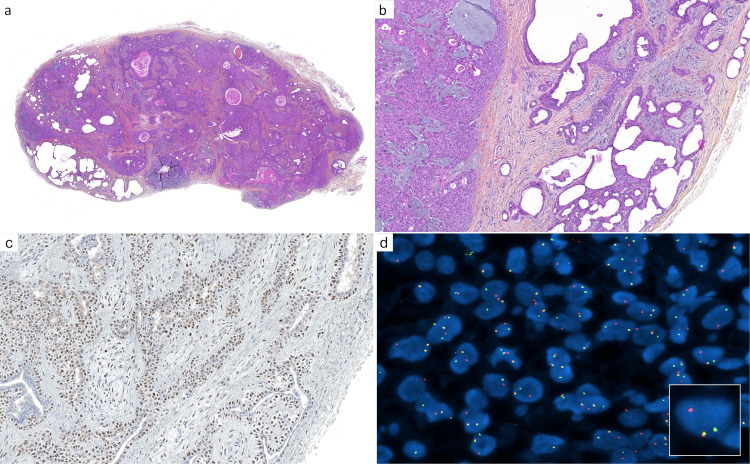
Cutaneous mixed tumor (chondroid syringoma): (**a**) cutaneous mixed tumor presenting as a circumscribed nodule, with cysts, ducts, nodules, and abundant stroma (×25); (**b**) higher magnification reveals different cell populations: myoepithelial aggregates (left), elaborated and cystic ductal structures (right), and mesenchymal cells associated with fibrous, fibromyxoid, and myxoid stroma (×100); (**c**) PLAG1 immunohistochemistry: diffuse nuclear staining is present, notably in the myoepithelial component (×200); (**d**) fluorescence in situ hybridization (FISH, break-apart probe) demonstrates a clonal *PLAG1* gene rearrangement (×1000).

General features: Cutaneous mixed tumor (chondroid syringoma, Figure 2) is a multiphenotypic epithelial, myoepithelial, and mesenchymal neoplasm.

The stroma is often fibrous, myxoid, chondroid, lipogenic, and osteogenic, imparting a distinctive morphology. Apocrine and eccrine-type cutaneous mixed tumors have been described. In addition to the sweat gland component, areas of follicular and/or matrical differentiation are frequent. Myoepithelioma is a related neoplasm lacking ductal differentiation (cf. myoepithelioma).

Related terminology: The homologous neoplasm in the salivary gland, breast, and lung is called pleomorphic adenoma.

Immunohistochemistry: Like adenoid cystic carcinoma, a cutaneous mixed tumor shows epithelial and myoepithelial differentiation but also a stromal component. The panel of antibodies to highlight its multiple cell populations includes SOX10, p63, PS100, calponin, SMA, EMA, c-KIT, CEA, and various cytokeratins [12,13,14]. Follicular differentiation stains with BerEP4 and PHDLA1. Although not evaluated in cutaneous cases, SOX10 is highly sensitive for the diagnosis of pleomorphic adenoma but lacks specificity [8,9]. A more specific marker is PLAG1 (clone 3B7), which is overexpressed in 87 to 100% of cutaneous mixed tumors, while rare cases show HMGA2 expression [30,31]. Since cutaneous mixed tumors of eccrine-type lack PLAG1 expression, it is unclear if cutaneous mixed tumors of apocrine and eccrine-type belong to the same spectrum until more studies are conducted [30]. PLAG1 is not expressed in soft tissue myoepithelioma lacking ductal structures [32], and has not been specifically tested in cutaneous myoepithelioma. Consistent with these findings, PLAG1 and HMGA2 show high sensitivity and specificity for the diagnosis of salivary pleomorphic adenoma and its related carcinoma [33,34,35]. The precise performance of PLAG1 and HMGA2 in skin cases requires larger studies.

Molecular biology: Recurrent rearrangements of PLAG1 are detected in 33% of cutaneous mixed tumors by FISH [31], which is a developmentally regulated zinc-finger proto-oncogene. PLAG1 activation through gene fusion typically occurs due to promoter swapping from various fusion partner genes that are ubiquitously expressed, such as *CTNNB1*, *LIFR,* and *CHCHD7.* This explains why pleomorphic adenomas of the salivary gland demonstrate a wide range of *PLAG1* and *HMGA2* fusions: *CTNNB1::PLAG1*, *LIFR::PLAG1*, *CHCHD7::PLAG1*, *TCEA1::PLAG1*, *HMGA2::FHIT*, *HMGA2::NFIB*, and *HMGA2::WIF1.* Although *NDRG1::PLAG1* and *TRPS1::PLAG1* fusions have been described in cutaneous cases [31], another study did not detect the fusions found in the salivary gland in cutaneous cases, suggesting different gene rearrangements or mechanisms [36]. To date, no *HMGA2* fusion has been reported in cutaneous mixed tumors. More studies are required to define more precisely the genetic landscape of cutaneous mixed tumors and, in particular, their relationship with myoepithelioma when their myoepithelial component is predominant (cf. myoepithelioma).

Discordant or controversial data: A PHF1::TFE3 fusion transcript was detected in one case morphologically diagnosed as malignant chondroid syringoma [37]. However, it remains to be determined whether this tumor actually belonged to the mixed tumor spectrum or was in fact a superficial ossifying fibro-myxoid tumor.

### 2.3. Cylindroma and Spiradenoma

**Figure 3:**
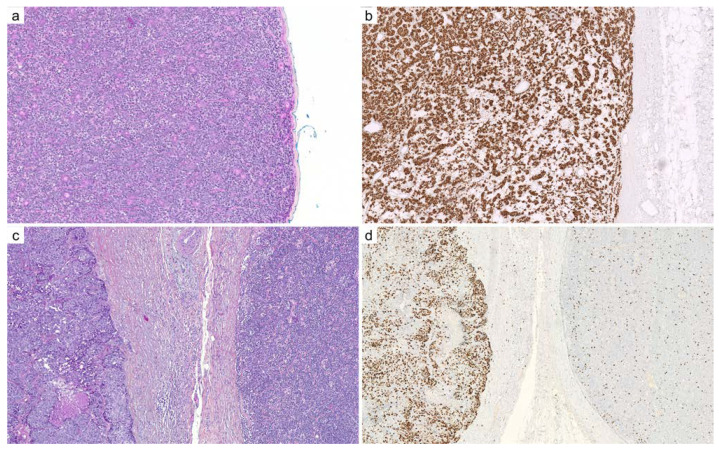
Transformation of spiradenoma into spiradenocarcinoma: (**a**) benign spiradenoma exhibits a dense nodular arrangement of myoepithelial and epithelial cells, intermingled with scattered lymphocytes (×100); (**b**) SOX10 immunohistochemistry diffusely and intensely stains the nuclei of spiradenoma, similar to adenoid cystic carcinoma (×100); (**c**) malignant transformation of a spiradenoma (right) into a spiradenocarcinoma (left): the malignant component demonstrates loss of architecture, pleomorphism, necrosis, cell crowding, increased mitotic activity, and loss of lymphocytic infiltrate (×100); (**d**) Ki67 immunohistochemistry: the labeling index is higher in the spiradenocarcinoma (left) compared to the precursor spiradenoma (right) (×100).

General features: Cylindroma and spiradenoma (Figure 3) are related epithelial and myoepithelial skin neoplasms, associated with activation of the NF-κB pathway, which is triggered by inactivation of *CYLD* or *ALPK1* mutations. Spiradenoma differs from cylindroma by its nodular architecture and lymphocytic infiltrate. On rare occasions, both neoplasms show a morphological and phenotypical overlap with adenoid cystic carcinoma. Most cases occur sporadically; however, some cases arise in the context of a predisposition syndrome known as Brooke–Spiegler syndrome. Multiple cylindromas, spiradenomas, trichoepitheliomas, and basal cell salivary gland tumors occur in this autosomic dominant disorder linked to *CYLD* mutations.

Related terminology: Homologous neoplasms in the salivary gland and breast are called basal cell adenoma (membranous variant of) and adenocarcinoma. Confusingly, the term ‘cylindroma’ has historically been used in the salivary gland to describe adenoid cystic carcinoma.

Immunohistochemistry: Cylindroma and spiradenoma staining patterns are similar to ACC [12,13,14]: SOX10 is intensely expressed, myoepithelial cells express calponin, SMA, and p63, while luminal cells express c-KIT (43%), EMA, CEA, and cytokeratins. MYB is expressed and cannot help differentiate from other sweat gland tumors [14]. Spiradenoma is infiltrated by CD3+ lymphocytes and CD1a+ Langerhans cells [38]. A distinctive claudin-4 staining pattern was found compared to poroma, syringoma, and hidradenoma in a single study [39]. The malignant transformation of spiradenoma is associated with an increase in Ki67 [40] and loss of MYB expression [41].

Molecular biology: Spiradenoma and cylindroma are related to oncogenic activation of the NF-κB pathway, triggered by mutually exclusive mutations of *ALPK1* and *CYLD* [29]. A wide range of *CYLD* mutations affect coding and splicing, resulting in inactivation of *CYLD* [42]. Most cases of cylindroma carry germline or somatic *CYLD* mutations compared to approximately a third of cases of spiradenoma [29]. Disruptive mutations in *DNMT3A* have also been described in cylindroma [29]. *ALPK1* mutations involve predominantly a hotspot in the alpha-kinase domain (p.V1092A) and are detected in 43% and 28% of spiradenomas and spiradenocarcinomas, respectively. Consistent with a model of progression and transformation from precursor spiradenoma, loss-of-function *TP53* mutations are restricted to spiradenocarcinoma [43].

Discordant or controversial data: MYB fusions are highly specific and are considered the hallmark of ACC [5,6,12,16,19,20], and have not been detected in *CYLD*-defective cylindroma [44]. A single study has reported that tumors diagnosed as *CYLD*-proficient dermal cylindroma demonstrated *MYB* fusions [28], which was not confirmed in a subsequent large-scale study [29] (cf. adenoid cystic carcinoma). Similar to adenoid cystic carcinoma, *CYLD*-defective cylindromas, however, demonstrate upregulation and overexpression of MYB and its target genes [44], suggesting the involvement of alternative mechanisms than MYB fusion [29]. *CYLD* mutations have recently been described in a subset of high-risk HPV-positive head and neck squamous cell and anal carcinomas, which show histological features close to cylindroma and have been designated as *CYLD*-mutant cylindroma-like basaloid carcinoma [45,46].

### 2.4. Hidradenoma 

**Figure 4:**
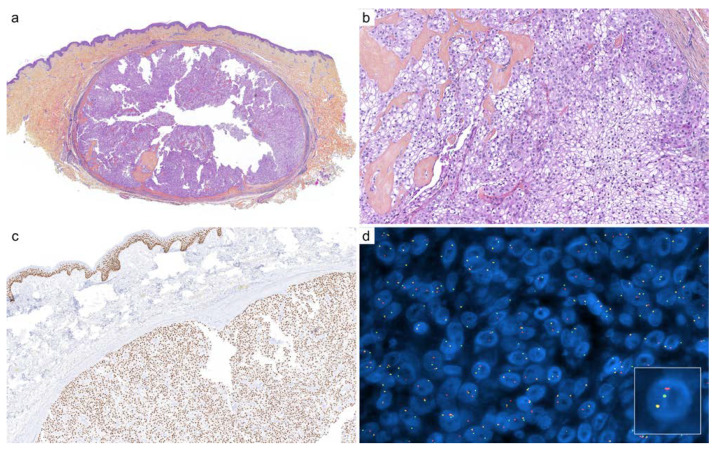
Cutaneous hidradenoma: (**a**) well-circumscribed nodular neoplasm (×25); (**b**) clear cell cytology and fibrous hyaline stroma (×200); (**c**) immunohistochemistry for p63 is usually intense and diffuse (×100); (**d**) fluorescence in situ hybridization (FISH, break-apart) demonstrates a clonal rearrangement of *MAML2* (×1000).

General features: Hidradenoma (Figure 4) is a dermal-based nodular neoplasm that exhibits clear, squamous, ductal, oxyphilic, and mucous-producing cells arranged in various proportions. Most cases demonstrate *MAML2* gene fusion. Malignant cases are called hidradenocarcinoma, a subset of which results from the transformation of a precursor hidradenoma.

Related terminology: The homologous neoplasm in the breast, salivary gland, lung, and pancreas is mucoepidermoid carcinoma.

Immunohistochemistry: Hidradenoma does not demonstrate a specific phenotype due to the combination of different types of cells. They express strongly and diffusely p40, p63, CK5/6, and AE1/AE3, while S100 and SMA are usually negative [47,48]. Immunostaining for EMA and CEA highlights ductal differentiation when present. Data on SOX10 expression are currently lacking, but, in our experience, SOX10 is constantly negative. Higher Ki67 and PHH3 labeling index and p53 expression have been reported in malignant cases [49].

Molecular biology: Half to 75% of the cases of hidradenoma have *CRTC1*::*MAML2*, and, more rarely, *CRTC3*::*MAML2* fusions [50]. Similarly, salivary gland mucoepidermoid carcinoma demonstrates 34–70% of *CRTC1*::*MAML2*, and, more rarely, *CRTC3*::*MAML2* fusions [51,52,53]. Hidradenocarcinomas also demonstrate *MAML2* rearrangement but with lower frequencies than their benign counterparts, while a subset carries *TP53, AKT1, PI3KCA* mutations, and Her2/neu amplification [54,55]. CREB-related transcriptional coactivator 1 (*CRTC1*) is located on chromosome 19 and activates cAMP-responsive element binding protein (CREB) signaling. Mastermind-like 2 (*MAML2*) is located on chromosome 11 and is associated with the Notch signaling pathway. EGFR overexpression has been found in the whole spectrum of cutaneous hiradenoma, namely hidradenocarcinoma, atypical hidradenoma, and hidradenoma [49]. However, a study investigating the amplification of *EGFR* in cutaneous cases lacked to demonstrate a clear correlation between the protein expression and the amplification of *EGFR*. Nonetheless, the EGFR signaling could still represent the mechanism of action of the *CRTC1::MAML2* fusion protein since the CRTC1 portion of the chimeric protein acts on AREG, which is a ligand of EGFR. In addition, some cases of salivary mucoepidermoid carcinoma that lack *CRTC1::MAML2* fusion rather demonstrate amplification and overexpression of EGFR and higher grade [56]. In this setting, therapies targeting the EGFR signaling axis are being investigated, such as Gefitinib [57]. Considering the current lack of data, the exact incidence of *MAML2* gene fusion in hidradenoma and hidradenocarcinoma requires more studies.

Discordant or controversial data: *EWSR1::POU5F1* fusion has been described in tumors diagnosed as hidradenoma in a single report [58] but has not been further confirmed [59]. A subsequent study has shown that this fusion defined a subset of myoepithelial tumors that can be mistaken for hidradenoma due to an extensive clear cell morphology [60] (*cf.* myoepithelioma).

### 2.5. Microcystic Adnexal Carcinoma

General features: Microcystic adnexal carcinoma (MAC) predominantly affects the sun-exposed area of the head and neck and has a high recurrence rate, slow growth, and low metastatic potential. MAC is an asymmetric and deeply infiltrative tumor that can present three components arranged in a vertical gradient, some of which may be lacking: small keratinizing cysts on the surface, solid aggregates in the center of the lesion, and, finally, deep tubular structures. As the histology of MAC in the superficial part of the skin resembles the histology of syringoma and basal cell carcinoma, an accurate diagnosis of MAC can be difficult on superficial biopsy. No specific molecular or immunohistochemical hallmark has yet been discovered.

Related terminology: Microcystic adnexal carcinoma is specific to the skin and lacks a homologous neoplasm in other organs. Solid carcinoma, reported in a series of 14 cases, may represent a variant of microcystic adnexal carcinoma [61]. A low-grade variant has recently been described as microcystic adnexal adenoma [62]. Hamartomatous lesions similar to MAC but with benign behavior have recently been reported in MALTA syndrome related to germline *MYH9* mutations [63].

Immunohistochemistry: Immunostains aim to differentiate between MAC, desmoplastic trichoepithelioma, basal cell carcinoma, and squamous cell carcinoma. In this setting, BerEP4, p63, and Ki67 can be used. BerEP4 shows negative or very infrequent staining in MAC, including the solid variant, while strong BerEP4 staining suggests basal cell carcinoma [61,64,65,66,67,68]. Immunostaining for p63 highlights scattered positive cells in the upper part of the neoplasm, but the deepest part of the MAC can be negative. On the contrary, p63 expression is diffuse in basal and squamous cell carcinoma [69,70]. Low-proliferation, i.e., Ki67 < 5%, favors MAC over basal and squamous cell carcinoma [68,71]. The luminal cells of the ductal structures are stained with CK7 and CEA, but this does not discriminate between other sweat gland neoplasms with ductal differentiation. PHLDA1 does not help to differentiate MAC from DTE, CK20 is usually negative [72], and SOX10 is almost always negative [73].

*Molecular biology:* Analysis of mutation burden, UV signature, mutational status of 400 cancer-relevant genes, and copy number variations showed few recurrent somatic mutations and, in most cases, a lack of an ultraviolet signature, a counterintuitive finding regarding the preferential occurrence of MAC in chronically sun-exposed skin [12]. The same study reported inactivating *TP53* mutations and frame-preserving indel mutations located in the kinase domain of *JAK1*, copy number alterations with gain of *JAK2*, *MAF*, *MAFB*, *JUN*, or *FGFR1,* and loss of *CDKN2A*. These data were consistent with another study in which sequencing indicated a mutation of *TP53* with loss of *CDKN2A* and *CDKN2B* in metastatic MAC [74]. Another study reported that, unlike basal cell carcinoma, Hedgehog signaling was not significantly altered in MAC [75]. Considering its lack of UV signature and since no oncogenic mutation was identified in one third of the cases in the largest study to date, more studies are required, including the detection of gene fusions, to reveal the genomic landscape of MAC.

### 2.6. Myoepithelioma

**Figure 5:**
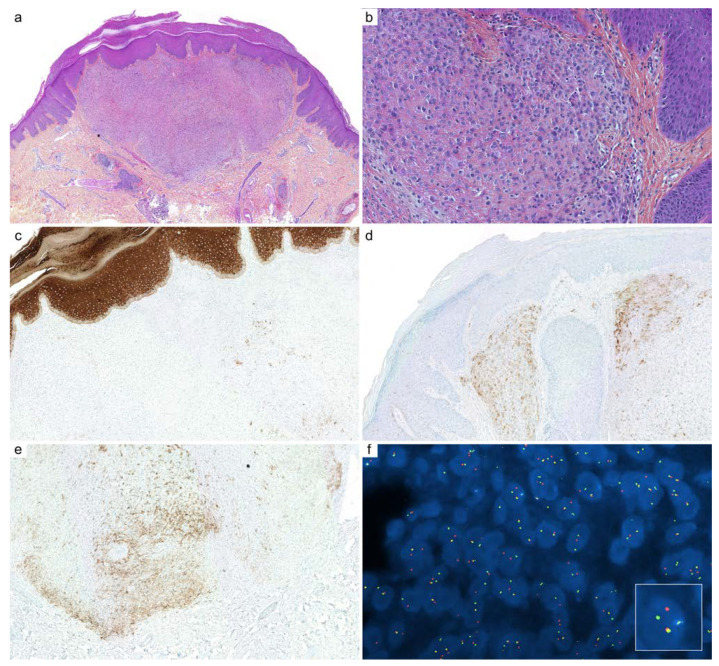
Cutaneous syncytial myoepithelioma: (**a**) this variant of myoepithelioma occurs predominantly in the superficial dermis (×25); (**b**) epithelioid and plasmacytoid cells are arranged in sheets with little or no intervening stroma (×200); (**c**) AE1/AE3 immunohistochemistry is most often negative or stains only scattered isolated cells (×100); (**d**) PS100 (×100) and (**e**) EMA immunohistochemistry stains various populations of cells (×100); (**f**) fluorescence in situ hybridization (FISH, break-apart) demonstrates clonal rearrangement of *EWSR1* gene (×1000).

General features: Myoepithelioma (Figure 5) is a rare neoplasm that occurs on the skin, soft tissue, salivary gland, and breast that is composed exclusively of myoepithelial cells. Half of extracutaneous cases are associated with various rearrangements of *EWSR1* or *FUS* [76]. Unlike mixed tumors, myoepitheliomas are defined by the lack of ductal structures. Half of cutaneous cases exhibit a distinctive skin-specific syncytial sheet-like pattern and harbor a *EWSR1::PBX3* fusion. Malignant cases are extremely rare.

Related terminology: The term myoepithelioma is used regardless of the organ considered. Depending on the classification, myoepithelial tumors are considered mesenchymal.

Immunohistochemistry: There is no specific immunostain for myoepithelial differentiation. Myoepitheliomas generally exhibit the expression of cytokeratins, S100 (70–80%), SOX10 (30–82%), calponin (86%), EMA (65%), GFAP (45%), SMA (35%), desmin (15%), and p63 (15%) [77,78,79]. Syncytial myoepitheliomas lack cytokeratins expression [80].

Molecular biology: *EWSR1* belongs to the TET family of proteins, a highly conserved group of multifunctional, RNA-binding proteins that includes fused in sarcoma (*FUS*). EWSR1 and FUS are related in both structure and function, are expressed in most cell and tissue types, and serve a multifunctional purpose and predominantly reside in the nucleus. Fusion proteins involving EWSR1 and FUS act as aberrant transcription factors (*EWSR1*/*FUS* providing the transcriptional activation domain and the other gene involved usually contributing the DNA-binding domain). While *EWSR1* gene fusions are lacking in cutaneous mixed tumors, many recurrent gene fusions involving the *EWSR1* and *FUS* genes have been reported in cutaneous and soft tissue myoepithelioma [60], with a proportion of 82% and 18%, respectively. *EWSR1*::*PBX3* fusions are more frequent in syncytial myoepithelioma compared to other cutaneous myoepitheliomas [80,81,82]. In extracutaneous myoepitheliomas, *EWSR1*::*POU5F1* and *EWSR1*::*PBX3* are the two most frequent fusions [76], while *FUS*::*KLF17*, *EWSR1*::*PBX1*, *EWSR1*::*ZNF444*, *EWSR1*::*KLF15*, *FUS*::*POU5F1*, *EWSR1*::*KLF17*, *EWSR1*::*FUS*, *EWSR1*::*ATF1*, and *EWSR1*::*VGLL1* are less frequent [60,76,83,84,85,86,87,88]. *POU5F1*-rearranged myoepitheliomas show a distinctive clear cell appearance that can be mistaken for hidradenoma, and *PBX1*-rearranged myoepitheliomas show sclerosis [60]. Loss of *SMARCB1* (INI1) is observed in a subset of malignant cases but has not been reported on the skin [89,90,91].

*Discordant or controversial data: EWSR1*::*POU5F1* translocation is frequent in a subset of myoepithelial tumors with frequent extensive clear cell morphology that can be mistaken for hidradenoma [60]. In this context and before the description of this fusion in clear cell myoepithelial neoplasms, *EWSR1*::*POU5F1* had been reported in tumors diagnosed as hidradenoma in a single report [58] but was not confirmed afterwards [59] (cf.: hidradenoma). PLAG1 fusions have been reported in myoepithelioma with ductal structures, and it is not yet clear whether these neoplasms are distinct from mixed tumors with a prominent myoepithelial component [92].

### 2.7. Poroma

**Figure 6:**
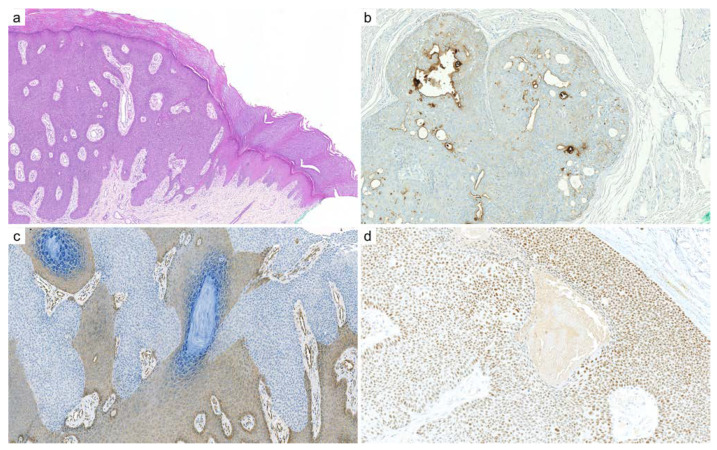
Eccrine poroma: (**a**) cutaneous eccrine poroma is composed of a population of poroid cells, with a sharp demarcation with adjacent epidermis, inconspicuous ducts, and numerous dilated vessels in the papillary dermis (×25); (**b**) CEA immunohistochemistry highlights the ductal differentiation (×200); (**c**) YAP1 (c-terminal) immunohistochemistry shows a clonal loss of expression in poroid cells compared to the adjacent epidermis, which suggest a *YAP1*-fusion (×200); (**d**) NUT immunohistochemistry demonstrates diffuse nuclear staining when a *NUTM1* gene fusion is involved, which is more frequent in the poroid hidradenoma variant (×200).

General features: Eccrine poroma (Figure 6) is morphologically characterized by a dual population of cells, namely poroid and cuticular cells, ductal differentiation, and demonstrates gene fusions involving *YAP1*, *MAML2*, and *NUTM1*.

Related terminology: The malignant counterpart of poroma is called porocarcinoma. The homologous malignant neoplasm in the salivary gland is called squamoid porocarcinoma. Dermal-based nodular poroma is called poroid hidradenoma [93].

Immunohistochemistry: The most recent immunohistochemical markers are YAP1 (clone D8H1X) and NUT (clone C52B1) [94,95,96]: loss of cytoplasmic expression of the c-terminal portion of YAP1 and/or aberrant nuclear expression of NUT both suggest the presence of an underlying gene fusion. YAP1 and NUT cannot discriminate between benign poroma and malignant porocarcinoma. Although preliminary data have suggested a high diagnostic specificity with respect to most differential diagnoses, the exact specificity of YAP1 is still being investigated. NUT immunohistochemistry is highly specific for *NUTM1*-rearranged cutaneous poroid neoplasms, and, among these, poroid hidradenoma shows the most frequent expression of NUT [95]. In general, NUT immunohistochemistry has 100% specificity and 87% sensitivity to detect *NUTM1* fusion [97]. NUT immunohistochemistry cannot discriminate between the different types of fusions, i.e., *YAP1::NUTM1*, *WWTR1::NUTM1*, and *BRD3/4::NUTM1* fusions, the latter occurring in NUT-midline carcinoma and exceptional cases of primary cutaneous carcinoma [98,99]. A higher Ki67 labeling index and aberrant expression of p53, RB, and p16 are more frequent in porocarcinoma [100,101,102]. Other stains, including CAE, EMA, c-KIT [103,104], and luminal cytokeratins, highlight ductal differentiation with variable sensitivity in poroma and porocarcinoma [105]. AE1/AE3 and cytokeratins 1, 5, 8, 10, and 14 are variably expressed within poroid and cuticular cells but lack specificity [106]. GATA3 [107], SOX10 [48], and GCDFP-15 [108] are usually negative.

*Molecular biology:* Recurrent *YAP1* rearrangements have been detected in 88% and 63% of cases of poroma and porocarcinoma, respectively [109], with subsequent reports detecting a *YAP1::MAML2* fusion in porocarcinoma [110] and in parotid gland squamoid porocarcinoma [111]. Yes1 associated transcriptional regulator (YAP1) is a transcriptional co-activator whose activity is controlled by the Hippo signaling pathway, regulating homeostasis and regeneration. YAP1 is also involved in cancer initiation, aggressiveness, metastasis, and therapy resistance [112]. Other fusions include *YAP1::NUTM1* and *WWTR1::NUTM1.* Consistent with immunohistochemical data, a single study reported that *YAP1::NUTM1* fusion was more common in benign and malignant poroid hidradenoma than in poroma and porocarcinoma [95]. *BRD3/4::NUTM1* fusion has been described in exceptional cases of primary or metastatic cutaneous adnexal carcinoma [98,99]. All the fusions harbor the N-terminal TEAD-binding domain of YAP1 or WWTR1 and the MAML2- or NUTM1-derived regions that interact with transcriptional coactivators CBP and p300 [109]. Porocarcinomas also carry mutations involving oncogenes and tumor suppressor genes, such as *CDKN2A*, *EGFR*, *ERBB2*, *FGFR3*, *HRAS*, *KRAS*, *NRAS*, *PIK3CA*, *TP53*, and *RB1* [109,113,114,115]. A recent cohort of morphologically diagnosed porocarcinoma reported a high mutational burden related to UV exposure, recurrent copy number alterations, including losses in chromosomal regions containing *BRCA2,* and deleterious alterations in multiple homologous recombination repair pathway components. Although gene fusions in this study were not tested to confirm diagnosis, these data encourage investigation of targeting the p53 axis, PARP inhibition, and immunotherapy in the treatment of porocarcinoma [116]. 

### 2.8. Secretory Carcinoma

**Figure 7:**
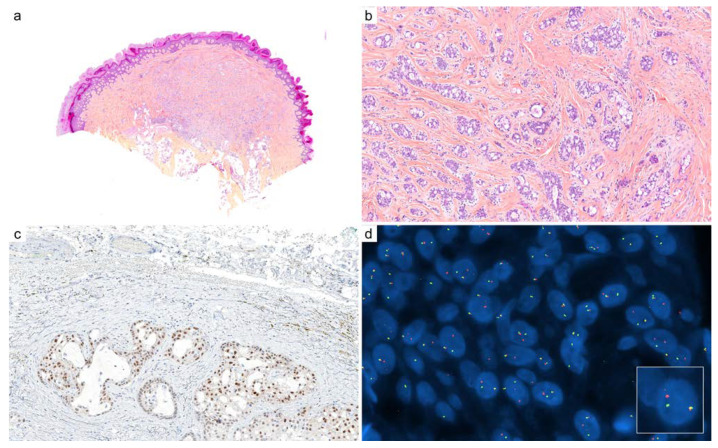
Cutaneous secretory carcinoma: (**a**) circumscribed intradermal neoplastic proliferation (×10); (**b**) intracytoplasmic secretory vacuoles and extracellular bubbly eosinophilic secretions (×100); (**c**) nuclear staining with panTRK immunohistochemistry is highly suggestive of *ETV6::NTRK3* fusion (×200); (**d**) fluorescence in situ hybridization (FISH, break-apart) demonstrates clonal and balanced rearrangement of *NTRK3* gene (×1000).

*General features:* Cutaneous secretory carcinoma (Figure 7) is a rare cutaneous *NTRK*-rearranged carcinoma that shows distinctive intracytoplasmic secretory vacuoles and bubbly extracellular eosinophilic secretions.

*Related terminology:* Secretory carcinoma occurs in the breast, salivary gland, lacrimal gland, lung, and skin. Cutaneous terminology includes ‘primary cutaneous mammary analogue secretory carcinoma’ (MASC) and ‘mammary-type secretory carcinoma of the skin’.

*Immunohistochemistry: NTRK* fusions can be detected by immunohistochemistry using a panTRK antibody (clone EPR 17341) with various performances and staining patterns, with a sensitivity up to 100% for secretory carcinoma [117,118,119]. When fusion involves *ETV6*, TRK immunohistochemistry is expected to stain the nucleus, while the cytoplasm and/or membrane are stained when alternative fusion partners are involved [120]. Other positive stains include SOX10, MUC4, S100, Mammaglobin, and STAT5A, while TTF1, DOG1, and p63 are negative [8,121,122]. 

*Molecular biology*: Independent of the organ considered, most cases of secretory carcinoma are associated with *ETV6::NTRK3* fusion [123,124]. The neurotrophic receptor tyrosine kinase (*NTRK*) family of genes includes *NTRK1*, *NTRK2*, and *NTRK3*, which regulate cell survival and differentiation. *NTRK* gene fusions have been shown to be actionable genomic events with the demonstration of highly efficacious outcomes with the use of TRK kinase inhibitors, such as larotrectinib and entrectinib [125]. An alternative *NFIX::PKN1* fusion has also been described [126]. Owing to its simple genomics, secretory carcinoma demonstrates low mutation burden and low copy number alterations [127]. The *ETV6::NTRK3* fusion is not specific and is detected in infantile fibrosarcoma, cellular mesoblastic nephroma, melanocytic Spitz nevus, pediatric astrocytoma, a subset of thyroid papillary carcinoma, leukemia, *NTRK*-rearranged mesenchymal spindle cell tumors, and inflammatory myofibroblastic tumors [126,128,129,130,131,132,133,134,135,136,137,138,139,140,141,142,143,144,145].

### 2.9. Tubular Adenoma and Syringocystadenoma Papilliferum

**Figure 8:**
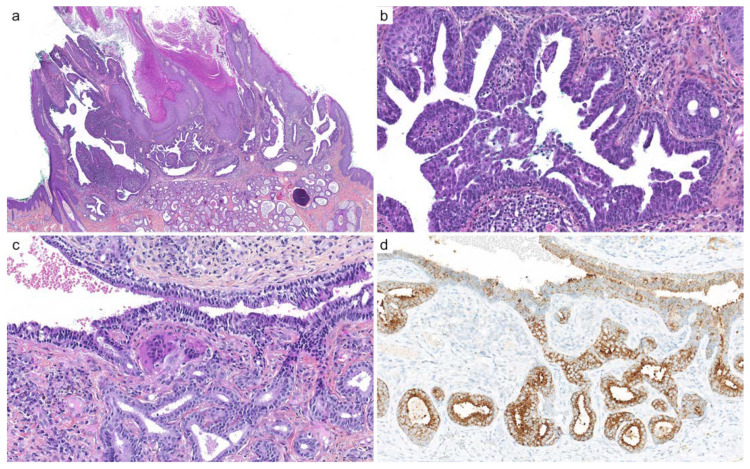
Syringocystadenoma papilliferum and tubular adenoma: (**a**) hybrid lesion with superficial syringocystadenoma papilliferum and dermal tubular adenoma arising on a nevus sebaceous, with verrucous change (×10); (**b**) syringocystadenoma papilliferum shows papillary projections, epithelial and myoepithelial cells, and numerous plasma cells in the adjacent dermis (×100); (**c**) tubular adenoma exhibits numerous ducts, tubes, and cysts with apocrine decapitation (×200); (**d**) BRAF p.V600E (clone VE1) immunohistochemistry intensely and diffusely stains the cytoplasm of neoplastic cells (×200).

General features: Tubular adenoma and syringocystadenoma papilliferum (SCAP, Figure 8) are related entities with an epithelial myoepithelial phenotype and tubulopapillary architecture [146]. Both are related to activation of mutations in the mitogen-activated protein kinase (MAPK) signaling pathway, affecting *BRAF*, *KRAS*, and *HRAS*. Both tubular adenoma and syringocystadenoma papilliferum can develop sporadically or more frequently on the genetic background of a cutaneous mosaic RASopathy, such as nevus sebaceus of Jadassohn [147,148].

Related terminology: The salivary and bronchus homologous neoplasm is called sialadenoma papilliferum.

Immunohistochemistry: Immunostains can be used successfully to detect cases with *BRAF* p.V600E mutation, with excellent sensitivity and specificity with the anti-BRAF p.V600E antibody (clone VE1) [149]. Epithelial myoepithelial differentiation of SCAP and tubular adenoma can be revealed by a panel including SMA, calponin, SOX10, and p63 to highlight the outer myoepithelial layer and luminal cytokeratins and EMA for the inner layer. Most cases are associated with plasma cells within the papillary axis, which can be stained with MUM1 and CD138.

*Molecular biology: BRAF* p.V600E mutations are detected in 50–64% of syringocystadenomas papilliferum and 66% of tubular adenomas, respectively. Tumors that arise in *nevus sebaceus* almost constantly harbor *HRAS* or *KRAS* mutations [150]. Activating mutations of *BRAF*, *HRAS*, and *KRAS* increase cell growth, differentiation, and survival. In sporadic tumors, *HRAS* p.G13R mutations are found in 7–26% of cases, while *KRAS* p.G12D mutations are less frequent [151,152]. DNA of HPV type 16 and 68 have been detected in SCAP of the perianal area and buttock, without HPV-related morphology [153]. Verrucous proliferations associated with SCAP demonstrate *BRAF* mutation in both the glandular and contiguous hyperplastic squamous epithelium [149,154,155]. Sialadenoma papilliferum exhibits the same morphology and carries *BRAF* p.V600E mutation in most cases, while reports of *RAS* mutations are currently lacking [156,157]. No data have been published on malignant SCAP, but various mutations have been reported in a case report of metastatic adenocarcinoma with papillary architecture, presumably originating from a SCAP that included *ARID2, CDKN2A, ERBB4, FAT1, FGF10, FGFR1, GNA11, KDM5C, MAGI2, NFKBIA, NOTCH1, PIK3CA, RAC1, RICTOR, SLIT2, TERT, TP53* mutations. More studies are needed to discover the molecular landscape of the malignant counterpart of SCAP. A case of digital papillary adenocarcinoma with *BRAF* p.V600E has been published but could represent a malignant form of tubular adenoma [158]. In this context, inhibitors of BRAF could be employed.

### 2.10. Endocrine Mucin-Producing Sweat Gland Carcinoma

**Figure 9:**
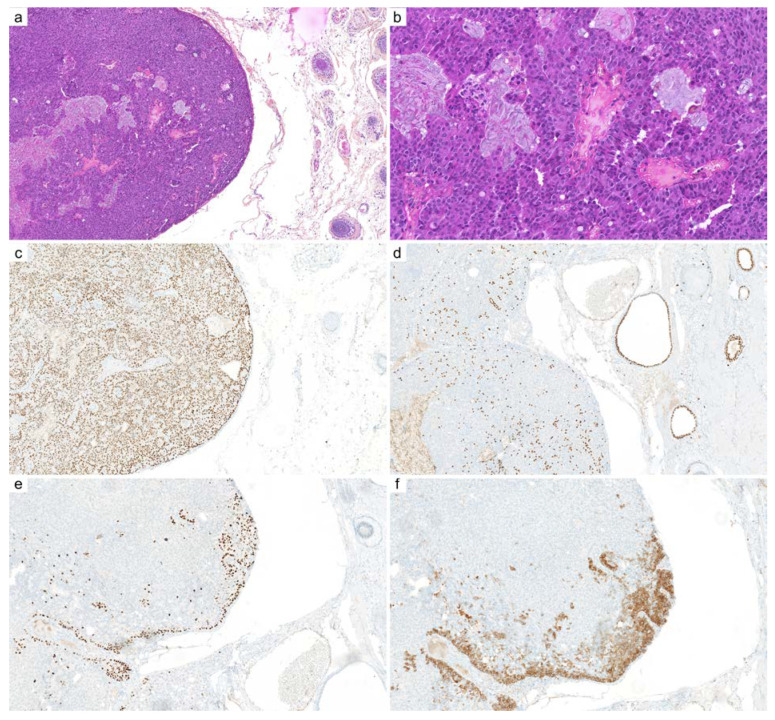
Histopathological findings immunohistochemistry of endocrine mucin-producing sweat gland carcinoma: (**a**) EMPSGC exhibits a well-circumscribed, nodular, sometimes cystic architecture (×100); (**b**) variable amount of intra- and extracellular mucin is present (×200); (**c**) immunohistochemistry for androgen receptor (AR) exhibits diffuse nuclear expression (×100); (**d**) SOX10 stains a variable proportion of cells (×100); the expression of neuroendocrine markers varies: INSM1 (×100) (**e**) and synaptophysin (×100) (**f**).

General features: Endocrine mucin-producing sweat gland carcinoma (EMPSGC, Figure 9) is a low-grade mucin-producing sweat gland carcinoma with a neuroendocrine phenotype and a predilection for the eyelids and periorbital skin [159,160].

Related terminology: The related homologous neoplasm is called solid papillary adenocarcinoma of the breast. Endocrine mucin-producing sweat gland carcinoma may represent a precursor of mucinous sweat gland carcinoma (neuroendocrine type) [159,160]. Low-grade neuroendocrine carcinoma of the skin/primary cutaneous carcinoid tumors might represent a variant or a closely related entity [161].

Immunohistochemistry: Endocrine mucin-producing sweat gland carcinoma (EMPSGC) expresses INSM1, synaptophysin (90%), chromogranin (71%), ER (98%), PR (96%), and AR [18,162]. Other positive stains include CK7, CK8, CK18, EMA, GCDFP15, WT1, Bcl2, MUC2, MUC4, RB1, SOX10, and MYB. Identification of a preserved myoepithelial layer expressing p63 supports the diagnosis [159,160,163,164,165,166]. 

Molecular biology: Identified mutations affect genes involved in DNA damage response/repair (*BRD4*, *PPP4R2,* and *RTEL1*), regulation of transcription/post transcriptional processing (*BRD4*, *RBM10*, *ZFHX3*, and *SMYD3*), and tumor suppressors (*BRD4*, *TP53*, *TSC1*, and *LATS2*) [167]. Other studies have not demonstrated relevant molecular drivers [164,168,169].

## 3. Main Clinical Settings to Use These Biomarkers and Their Limits

Although some of these biomarkers are directly linked to a specific diagnosis, other markers are associated with a whole spectrum. In fact, demonstrating a rearrangement of *MYB, MYBL1*, *PLAG1*, *HMGA2*, *NTRK3,* and a *BRAF* mutation in the appropriate morphological context is diagnostic of adenoid cystic carcinoma, cutaneous mixed tumor, secretory carcinoma, syringocystadenoma papilliferum, and tubular adenoma. On the other hand, the demonstration of fusion of *YAP1*, *NUTM1*, *MAML2*, *EWSR1*, *FUS*, and mutation of *CYLD* and *ALPK1* points to the poroid, hidradenomatous, myoepithelial, cylindromatous, and spiradenomatous categories of tumors, and these alterations do not predict prognosis or malignancy, which is based on other characteristics, such as morphology and proliferation. Other alterations are used for diagnostic and theranostic purposes, such as the *BRAF* p.V600E mutation and the fusion of *NTRK3*.

Some cases still lack any detectable molecular or immunohistochemical hallmark, and there is not a single answer to this question: variation in the sensitivity of the molecular techniques used, unreported gene fusion, alternative molecular mechanisms, and misinterpretation of pathological findings may contribute to the variation in prevalence of observed anomalies. The exact prevalence of these molecular anomalies is still unknown given the lack of data and large studies that include bona fide cases.

## 4. Other Sweat Gland Tumors Lacking Recent Data

Numerous other sweat gland tumors and related carcinomas still lack extensive molecular or immunohistochemical data, notably histiocytoid carcinoma, papillary digital adenocarcinoma, polymorphous sweat gland carcinoma, squamoid eccrine ductal carcinoma, mucinous carcinoma, apocrine carcinoma, and adnexal adenocarcinomas without other specification (NOS). This lack of data is the consequence of the combination of their rarity and diversity, which prevents large-scale studies from focusing on their molecular background. Networks of pathologists with common and robust diagnostic criteria, collaborative work, and collections of these rare neoplasms are of utmost importance to improve their diagnosis and allow proper future research [1,170,171].

## 5. Conclusions

The molecular landscape of cutaneous adnexal tumors has changed dramatically in the past few years. The addition of robust biomarkers in an integrated molecular–pathological approach is more likely to positively impact the reproducibility of diagnoses. Due to their great variety, rarity, complex terminology, and overlapping microscopic and phenotypic features, the pathological diagnosis of rare cutaneous neoplasms is challenging. However, these novel molecular and immunohistochemical hallmarks are expected to improve overall diagnostic accuracy due to their high specificity when integrated into an appropriate clinical and pathological context. Ultimately, since the entities will be systematically validated with a specific hallmark, standardized diagnoses will pave the way for future studies investigating the prognosis and therapeutic management of these rare cutaneous tumors.

## Figures and Tables

**Table 1:** Most recent immunohistochemistry used for the diagnosis of skin adnexal neoplasms with sweat gland differentiation.

Diagnoses Considered	Antibody	Staining Pattern	Reported Positivity
Adenoid cystic carcinoma, cylindroma, and spiradenoma	SOX10 MYB	nuclear nuclear	73–100% 70–90%
Cutaneous mixed tumor	PLAG1 HMGA2	nuclear nuclear	87–100% unknown
Poroma, porocarcinoma, poroid hidradenoma	YAP1 NUT	cytoplasmic (loss) nuclear	58–80% 29–32%
Secretory carcinoma	panTRK	nuclear	100%
Syringocystadenoma papilliferum and tubular adenoma	BRAF^V600E^	cytoplasmic	50–64%
Endocrine mucin-producing sweat gland carcinoma	INSM1	nuclear	100%

**Table 2:** Summary of the most frequent molecular alterations in sweat gland neoplasms.

Diagnosis	Molecular Alteration	Frequency (%)
Adenoid cystic carcinoma	*MYB::NFIB* fusion *MYBL1::NFIB* fusion	73–83% 20–23%
Cutaneous mixed tumor	*PLAG1* fusion *HMGA2* fusion	33% unknown
Cylindroma	*CYLD* inactivation	near 100%
Spiradenoma	*CYLD* inactivation *ALPK1* p.V1092A mutation	29% 43%
Spiradenocarcinoma	*CYLD* inactivation *ALPK1* p.V1092A mutation	8% 33%
Hidradenoma	*CRTC1::MAML2* fusion *CRTC3::MAML2* fusion	50–75% rare
Hidradenocarcinoma	*CRTC1::MAML2* fusion	unknown
Myoepithelioma	*EWSR1* fusion *FUS* fusion	82% 18%
Poroma	*YAP1* fusion *NUTM1* fusion	88% 17–55%
Porocarcinoma	*YAP1* fusion *NUTM1* fusion	8–63% 11–54%
Secretory carcinoma	*ETV6:NTRK3* fusion	near 100%
Syringocystadenoma papilliferum and tubular adenoma	*BRAF* p.V600E mutation *HRAS* p.G13R mutation *KRAS* p.G12D mutation	50–64% 7–26% rare
